# Effects of Antibiotics on the Growth and Physiology of Chlorophytes, Cyanobacteria, and a Diatom

**DOI:** 10.1007/s00244-016-0305-5

**Published:** 2016-08-09

**Authors:** Jiahua Guo, Katherine Selby, Alistair B. A. Boxall

**Affiliations:** Environment Department, University of York, Wentworth Way, Heslington, York, YO10 5NG UK

## Abstract

The occurrence of antibiotics in surface waters has been reported worldwide with concentrations ranging from ng L^−1^ to low µg L^−1^ levels. During environmental risk assessments, effects of antibiotics on algal species are assessed using standard test protocols (e.g., the OECD 201 guideline), where the cell number endpoint is used as a surrogate for growth. However, the use of photosynthetic related endpoints, such as oxygen evolution rate, and the assessment of effects on algal pigments could help to inform our understanding of the impacts of antibiotics on algal species. This study explored the effects of three major usage antibiotics (tylosin, lincomycin, and trimethoprim) on the growth and physiology of two chlorophytes (*Desmodesmus subspicatus* and *Pseudokirchneriella subcapitata*), a cyanobacteria (*Anabaena flos-aquae*), and a diatom (*Navicula pelliculosa*) using a battery of parameters, including cell density, oxygen evolution rate, total chlorophyll content, carotenoids, and the irradiance–photosynthesis relationship. The results indicated that photosynthesis of chlorophytes was a more sensitive endpoint than growth (i.e., EC_50_ derived based on the effects of tylosin on the growth of *D. subspicatus* was 38.27 µmol L^−1^ compared with an EC_50_ of 17.6 µmol L^−1^ based on photosynthetic rate), but the situation was reversed when testing cyanobacteria and the diatom (i.e., EC_50_ derived based on the effects of tylosin on the growth of *A. flos-aquae* was 0.06 µmol L^−1^; EC_50_ 0.33 µmol L^−1^ based on photosynthetic rate). The pigment contents of algal cells were affected by the three antibiotics for *D. subspicatus*. However, in some cases, pigment content was stimulated for *P. subcapitata*, *N. pelliculosa*, and *A. flos-aquae*. The light utilization efficiency of chlorophytes and diatom was decreased markedly in the presence of antibiotics. The results demonstrated that the integration of these additional endpoints into existing standardised protocols could provide useful insights into the impacts of antibiotics on algal species.

**Electronic supplementary material:**

The online version of this article (doi:10.1007/s00244-016-0305-5) contains supplementary material, which is available to authorized users.

Antibiotics are used in human and veterinary medicine and, in some regions, also are employed as farm animal feed additives for agricultural purposes (Boxall 2004). Antibiotics can be released to the aquatic environment at different stages in their life-cycle. For antibiotics used in humans, the main route of emission will be to the wastewater system and then into surface waters (Boxall 2004). For veterinary antibiotics, compounds can be released directly to aquatic systems when they are used in aquaculture products or are excreted or washoff from pasture animals in streams. Antibiotics that are released to the soil environment either directly or during manure/slurry or sludge application can subsequently be transported to surface waters via runoff and drainage (Boxall 2004).

The presence of antibiotics in surface water has been reported worldwide. For example, concentrations of trimethoprim have been reported to range from less than 3.4 × 10^−5^ µmol/L in UK surface waters to 0.0061 µmol/L in the United States (Ashton et al. 2004; Kolpin et al. 2002). The presence of lincomycin in surface water has been recorded from less than 2.46 × 10^−6^ µmol/L to 0.0018 µmol/L in U.S. surface waters (Monteiro and Boxall 2010). The maximum occurrence of tylosin was found at 5.46 × 10^−5^ µmol/L downstream of agricultural land in the United States (Boxall et al. 2011).

While the environmental effects of antibiotics on several aquatic organisms across three trophic levels (fish, invertebrates, and algae) have been reported (Santo et al. 2010; Crane et al. 2006; Guo et al. 2015), studies have demonstrated that algae are particularly sensitive to antibiotics compared with other two trophic levels. For example, after exposure to lincomycin, the 3-days median effective concentration values (EC_50_) for the chlorophyte *P. subcapitata* was 0.16 µmol L^−1^, which is two orders of magnitude lower than effect concentrations for crustacea (*Daphnia magna*) and zebrafish (*Danio rerio)* with a 1-day median lethal concentration (LC_50_) 52.32 µmol L^−1^ being observed for the daphnids and a 2-days no observed effect concentration (NOEC) of 2257 µmol L^−1^ being observed for zebrafish (Isidori et al. 2005). Studies to date into the effects of antibiotics on algae generally have assessed impacts on the growth of a range of algal species and communities (Wilson et al. 2003; Cleuvers 2003; DeLorenzo and Fleming 2008; Guo et al. 2016) using biomass (i.e., cell number) as the endpoint, as suggested by standard bioassay protocols such as the Organisation for Economic Co-operation and Development (OECD 201 guideline) (OECD 2011), which includes the standard methods to evaluate the effects of a chemical on the growth of an algal species.

While antibiotics are designed to interact with receptors in pathogenic bacteria, the fact that similar receptors and/or pathways also might be conserved in algal species means that the exposure to antibiotics in the natural environment could pose a potential threat to the growth of algae (Boxall 2004). Macrolide antibiotics could inhibit the growth of eukaryotic species by interfering with the protein and enzyme synthesis involved in the photosynthesis process (Liu et al. 2011). For example, approximately 30 proteins of cytochrome bf complex, which are the important component for the membrane in the thylakoid of algae, are involved in photosynthesis I and II pathways. The macrolide (e.g., erythromycin) has been found to reduce the membrane content by interfering with the electron transport from PS II to PS I and reducing the size of the receptor-side of PS II (Liu et al. 2011). Ribulose bisphosphate carboxylase (Rubisco) is an essential enzyme to catalyse the addition of CO_2_ to ribulose-1,5-bisphosphate (RuBPCase) during the Calvin Cycle in the algal photosynthesis (Cooper 2000). Macrolides could adversely influence the activity of rubisco and further inhibit the synthesis and activity of the RuBPCase in algae (Liu et al. 2011).

At present little is known about the direct effects of antibiotics on light-harvesting pigment synthesis and light utilization efficiency, although they are the prerequisites for proceeding photosynthesis metabolism in algae and cyanobacteria. The energy of sunlight is captured by the light-harvesting pigments such as chlorophyll and carotenoids in the wavelength range of 700–400 nm. While light utilization efficiency involves a variety of complex processes, it could be readily investigated by exploring the relationship between the irradiance and photosynthetic rate (Bahrs et al. 2013). As algal species play a critical role in key ecosystem functions, such as primary production (e.g., provide biomass to higher trophic levels via food chain) and nutrient transformation (e.g., nitrogen fixation), antibiotics could be adversely impacting aquatic ecosystems (Guo et al. 2015). While photosynthetic endpoints, such as short-term oxygen evolution rate and pigment synthesis (i.e., chlorophyll content), have been used in a range of studies investigating the effects of external stressors on algal photosynthetic process, researchers have primarily focused on the impacts of stressors such as herbicides (Xia 2005; Wong 2000). However, no antibiotic studies have attempted to compare the sensitivity of algal photosynthesis related endpoints (e.g., oxygen evolution rate) and growth (i.e., cell counts). For the effect assessment of antibiotics on algal species, an understanding of the endpoint sensitivity for species from the chlorophyte, cyanobacteria, and diatom groups would be valuable to understand the potential influence of antibiotics on ecosystems.

The objectives of the present study were: (1) to compare the sensitivity of photosynthesis-related endpoints (i.e., oxygen evolution rate) and growth (i.e., cell counts) following 4-days exposure to antibiotics; and (2) to evaluate the inhibitory effects of the antibiotics on the algal physiology including light-harvesting pigment synthesis and light utilization efficiency. The work focused on three antibiotics tylosin, lincomycin, and trimethoprim, which have been highly ranked in a recent prioritisation study of pharmaceuticals in the natural environment where they all demonstrated risk scores greater than one, based on ecotoxicity to algae (Guo et al. 2015). Four species, as suggested by the OECD 201 guideline, were studied, including two chlorophytes (*Pseudokirchneriella subcapitata* and *Desmodesmus subspicatus*), a cyanobacteria (*Anabaena flos-aquae*), and a diatom (*Navicula pelliculosa*). These species previously have been shown to be sensitive to these three antibiotics in a recently sensitivity comparison study (Guo et al. 2016).

## Method

### Chemicals

Tylosin tartrate (referred to as tylosin, 86.4 %) (CAS-no. 1405-54-5), lincomycin hydrochloride (referred to as lincomycin, ≥95 %) (CAS-no. 859-18-7), trimethoprim (≥98 %) (CAS-no. 738-70-5), and potassium dichromate (≥99.8 %; used as reference substance) were purchased from Sigma-Aldrich. Ammonium acetate and formic acid (≥95 %) as analytical reagent grade were purchased from Fisher Scientific UK and Sigma-Aldrich, respectively. Acetonitrile, methanol, and water (HPLC Gradient grade) were purchased from Fisher Scientific UK.

### Algae Culture

*Pseudokirchneriella subcapitata* (CCAP 278/4), *D. subspicatus* (CCAP 258/137), *A. flos-aquae* (CCAP 1403/13A), and *N. pelliculosa* (CCAP 1050/9) were supplied by the Institute of Freshwater Ecology (Culture Collection of Algae and Protozoa, UK). *P. subcapitata* and *D. subspicatus* were cultured in Kuhl medium, pH 6.8 (Kuhl and Lorenzen 1964); *A. flos-aquae* was grown in Jaworski’s Medium (JM), pH 7.8 (CCAP 2014); *N. pelliculosa* was grown in Enriched Seawater-Artificial Water (ESAW) and f/2 medium, pH 8.2 (Berges et al. 2004). Triplicate cultures of each species were initiated by adding 100 mL of medium and 1 mL of algal stock to a 250-mL Erlenmeyer flask. The four species were grown in an incubator with 24-h illumination (76 µmol m^−2^ s^−1^) with continuous shaking [100 cycles per minute (cpm)] at a fixed temperature (20 ± 2 °C). All flasks involved were washed with Decon 90, rinsed with hydrochloric acid (50 mM), and then autoclaved (at 121 °C for 30 min) before use. Cell numbers of the cultured species were counted daily with a haemocytometer under a microscope, and growth curves (cell density over time) were plotted to find the logarithmic phase (usually during 2–4 days cultivation). The algal stocks were subcultured on a weekly basis.

### Procedure for the Growth Inhibition Test

Growth inhibition tests were performed following the OECD Guideline 201 (OECD 2011). All glassware and stoppers used in the tests were autoclaved at 121 °C for 30 min before use. Triplicates of six concentrations of each antibiotic and a negative control were prepared in the corresponding culture medium solution. After addition of the antibiotic, samples sterilized by filtration (pore size 0.2 µm) were added into a 25-mL vial, and precultured algal cells grown in the logarithmic phase were inoculated into the vial to obtain 15-mL solution with an initial density 5 × 10^5^ cells mL^−1^. Following the inoculation, these vials were capped with air-permeable stoppers made of cotton and muslin. All operations were undertaken in a sterilized chamber, and the vials were then incubated for 4 days under the same conditions as the cultures.

Cell density in each sample was measured at 24-h intervals using UV–Visible spectrophotometry. Cell density was calculated from a calibration curve of known cell density counted by a haemocytometer against adsorption measured by an ultraviolet and visible (UV–Vis) spectrophotometry (*R* ^2^ > 0.999) for each test species. Measurement of turbidity (adsorption) using a spectrophotometer set at a selected wavelength is a reliable method to determine cell density (ABO 2013). Each algal culture was diluted and scanned over the 600–800 nm range. The wavelengths with the highest absorbance were selected for experiments. *P. subcapitata* was detected at a wavelength of 750 nm and *D. subspicatus*, *A. flos-aquae,* and *N. pelliculosa* at a wavelength 682 nm. Growth inhibition of each alga was calculated from the yield of algal cell density in each treatment after 4-days exposure. Yield is calculated as the cell density at the end of the test minus the starting cell density for each single vessel of controls and treatments. The percent inhibition in yield (% *I*_y_) was calculated by Eq. 1 (OECD 2011):

1 $$\% I_{\text{y}} = \left( {Y_{\text{C}} {-}Y_{\text{T}} } \right)/Y_{\text{C}} \times 100$$

where % *I*_y_ is the percentage inhibition of yield; *Y*_C_ the mean value for yield in the control group; and *Y*_T_ is the value for yield for the treatment replicate.

Two-step experiments including range-finding and determination were conducted in growth inhibition tests. Initial range-finding studies, which consisted of six concentrations (maximum 93.79, 225.73, and 344.45 µmol L^−1^ for tylosin, lincomycin, and trimethoprim, respectively) in geometric series and a negative control, were used to estimate the median effective concentration values (EC_50_) range. Six concentrations around the estimated EC_50_ in geometric series and a negative control were then selected for use in the definitive study. Each treatment and negative control had three replicates.

The prepared concentrations of antibiotics before the test were confirmed by chemical analysis. Samples with the highest and lowest concentrations were analysed again after the test to determine the antibiotic stability. Recovery was defined as the antibiotic concentration in algal solution after 4-days exposure compared with the initial concentration. For algal toxicity tests with chemical recoveries more than 80 % after the 4-days period, initial nominal concentrations were applied to derive the concentration–response curve. In several algal toxicity tests, the recoveries of antibiotics in the highest and lowest test concentrations were less than 80 % after the 4-days test. In these cases, it was assumed that dissipation followed first-order kinetics (Eq. 2) and a dissipation rate constant (*k*) was estimated. The k was then applied in Eq. 3 to estimate the time-weighted average concentration (TWAC) over *t* days (where *t* = 1, 2, 3, 4). By comparing the TWAC with the nominal concentration, a correction factor was then obtained for use in the concentration response analyses. Observations from the low concentration recovery tests were used for correcting the three lowest concentrations used in the ecotoxicity study, whereas concentrations for the high concentration recovery were used for correction of the three highest concentrations.

2 $$C_{t} = C_{0} \times e^{ - kt}$$

3 $$C_{\text{avet}} = C_{0} \times \left( {1 - e^{ - kt} } \right)/kt$$

where *C*_0_ is the initial concentration (µmol/L); *C*_*t*_ the concentration at the *t* day (µmol/L); *C*_avet_ the average concentration over *t* days (µmol/L); *k* the rate constant (day^−1^) and *t* is the time (day; Boesten et al. 1997). Based on these modified exposure concentrations and percentage inhibition of yield (% *I*_y_; Eq. 1), concentration–response curves were obtained by fitting regression analysis of sigmoidal functions (sigmoid, logistic, weibull, gompertz, hill, and chapman equations) embedded in the Sigma plot software version 12.0. The best fitting model (highest coefficient of determination *R* ^2^) was used for calculating median effective concentration values (EC_50_) based on growth as the endpoint.

### Photosynthetic Oxygen Evolution

After 4-days exposure to the antibiotics, algae from the growth studies were taken and the oxygen evolution rate was determined using a DW2 Oxygen Electrode Chamber (Hansatech Instruments Limited, UK). The measurement was firstly performed for 10 min under dark conditions at 20 °C to give the respiration rate (*R*). A 15 min measurement under illumination of 76 µmol m^−2^ s^−1^ actinic light intensity was then performed to give the photosynthesis rate (*P*_*n*_). The gross photosynthesis rate (*P*_g_) was the sum of these two processes. The percent inhibition in gross photosynthesis (% *I*_P_) was calculated by Eq. 4:

4 $$\% I_{\text{P}} = \left( {P_{\text{C}} {-}P_{\text{T}} } \right)/P_{\text{C}} \times 100$$

where % *I*_P_ is the percentage inhibition in gross photosynthesis; *P*_C_ the mean value for gross photosynthesis in the control group; and *P*_T_ is the value for gross photosynthesis for the treatment replicate. Based on the modified exposure concentrations and percentage inhibition in gross photosynthesis (% *I*_P_; Eq. 4), concentration–response curves of photosynthesis plotted by using Sigma plot 12.0 were used to derive EC_50_ based on the photosynthesis endpoint.

### Photosynthetic Pigment Content

 After 4-days exposure in the growth studies, 5 mL of each treated sample was first filtered using a 25-mm fibre filter (Pall Corporation, UK). Afterwards, the filter was put into a vial with 5 mL of methanol, and kept for 24 h in a spark-free fridge to extract photosynthetic pigment content. Chlorophyll a and b were estimated using the Wellburn coefficient equation (Eqs. 5 and 6; Wellburn 1994) and total chlorophyll content was the sum of them. The total carotenoid were estimated using the Lichtenthaler equation (Eq. 7). Absorbance values (A_470_, A_653_, and A_666_) were measured by UV–Vis spectrophotometry at 470, 653 and 666 nm. For each experimental measurement, the result was corrected for cell density.

5 $${\text{Chlorophyll a}}\,\left( {{\text{mg L}}^{ - 1} } \right) = 1 5. 6 5 {\text{A}}_{ 6 6 6} - 7. 3 4 {\text{A}}_{ 6 5 3}$$

6 $${\text{Chlorophyll b}}\,\left( {{\text{mg}}\,{\text{L}}^{ - 1} } \right) = 2 7.0 5 {\text{A}}_{ 6 5 3} - 1 1. 2 1 {\text{A}}_{ 6 6 6}$$

7 $${\text{Total carotenoids}}\,\left( {{\text{mg L}}^{ - 1} } \right) = \left( { 1000{\text{ A}}_{ 4 70} - 4 4. 7 6 {\text{ A}}_{ 6 6 6} } \right)/ 2 2 1$$

### Irradiance–Photosynthesis (I–P) relationship measurement

Triplicates of a negative control and a treatment at the EC_50_ of each antibiotic, based on the gross photosynthesis endpoint, were prepared. Algae were then innoculated into the control and antibiotic treatments and exposed for 4 days after which gross photosynthesis rate (*P*_g_) of the samples was measured under five different light intensities: 76, 150, 300, 450, and 600 µmol m^−2^ s^−2^. *P*_g_ for each light intensity was measured following the procedures in “Photosynthetic Oxygen Evolution” section. Bar charts of gross photosynthesis rate (*P*_g_) versus light intensity were plotted to analyse the effects of antibiotics on the algal light utilisation efficiency.

### Chemical Analysis Procedures

Concentrations of the antibiotics in the exposure solutions were confirmed using high performance liquid chromatography (HPLC) using an Agilent 1100 with C18 Supelco Discovery column (15 cm × 4.6 mm × 5 µm). Analytical methodologies were described in detail in Guo et al. (2016). In brief, tylosin and trimethoprim were analysed using a 24-min gradient method. The mobile phase consisted of methanol (A) and a buffer (B) (50 mM ammonium acetate plus 0.01 % formic acid, pH 6.5 adjusted with 2.5 % ammonium solution). The gradient was as follows: 5-min equilibration at a 10:90 ratio (A:B); 2 min at 50:50; 20 min at 90:10; and 2 min at 10:90. A retention time of 13 min with a flow rate of 1 mL min^−1^ and detection wavelength of 280 nm was used for tylosin and 6.4 min, 1 mL min^−1^, 238 nm was used for trimethoprim. Lincomycin was analysed by an isocratic method using 0.1 % formic acid plus acetonitrile at a ratio 75:25 with a retention time of 4 min, flow rate of 1.2 mL/min and a detection wavelength of 196 nm. Quantification was performed from a calibration curve constructed from standards of each antibiotic and relating concentration to peak area. For measuring low concentration solutions (<0.28 µmol/L) of tylosin and lincomycin (<0.68 µmol/L) for the cynobacterial tests, solid phase extraction (SPE) was used to concentrate the samples prior to analysis. Oasis HLC 3cc extraction cartridges were used and were purchased from Waters (UK). The SPE procedures were as follows: cartridge conditioning was undertaken by adding 6 mL of methanol followed by 6 mL of water. The sample (100 mL) was then loaded onto the SPE. The cartridges were then rinsed with 6 mL of water and eluted using 6 mL of methanol. Eluates were then concentrated, by evaporation with nitrogen in a fume hood, to dryness before being taken up in 1 mL of methanol.

### Statistical Methods

Significant differences between oxygen evolution rate and pigment content in treatments and controls were determined using the One way ANOVA Dunnett test with *p* < 0.05. Two-way ANOVA Tukey test was used for the irradiance–photosynthesis relationship study, where all data passed the test for normality. To explore whether pH values were significantly different for media at the start and at the end of test, pH values of controls (*n* = 3) in each algal test were compared with treated samples using Tukey’s test (*p* < 0.05)

## Results and Discussion

### Analysis of Chemical Stability, pH Variation, and Reference Substance

**Fig. 1:**
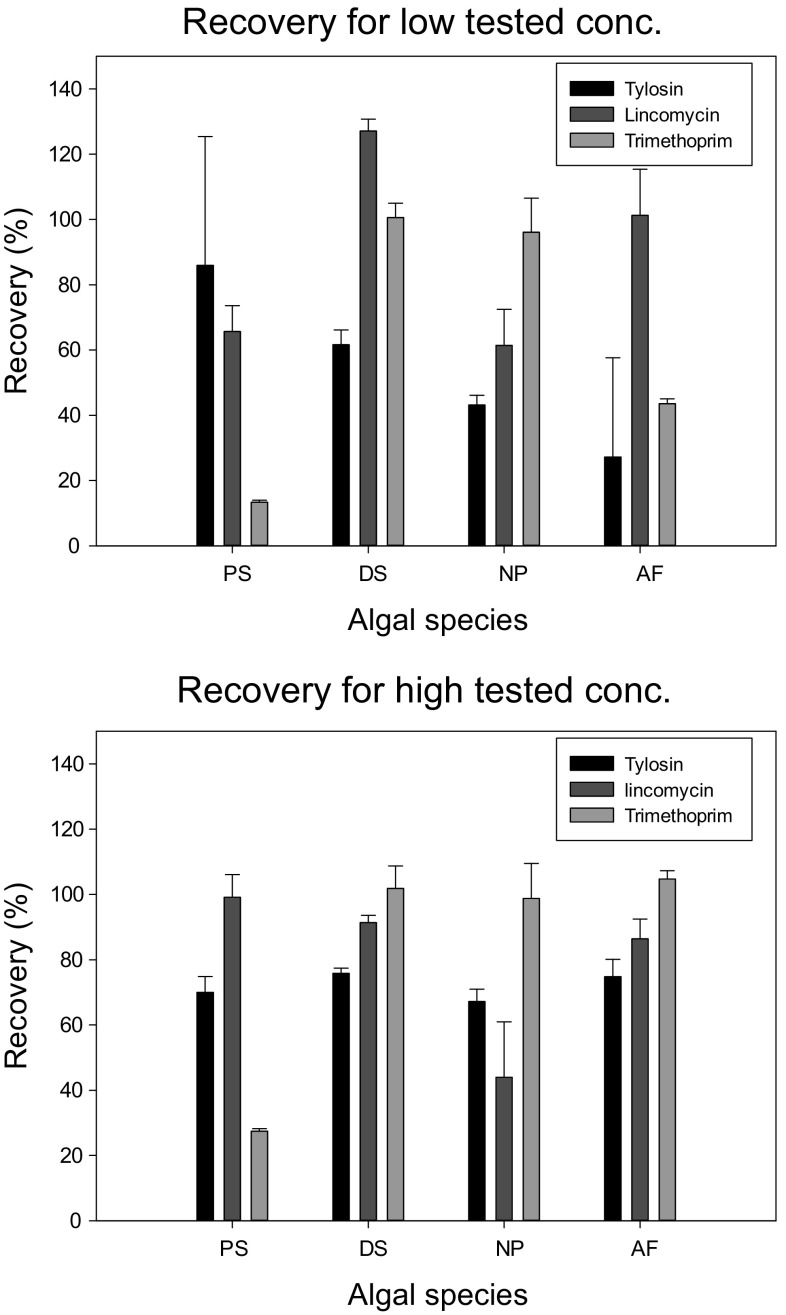
The amount (expressed as a % of the starting concentration) of the three study antibiotics remaining in the exposure media used in the growth samples (data are shown for the lowest and highest test concentration for each study). Data represent mean ± SD (*n* = 3). Antibiotic recoveries for *A. flos-aquae* were extracted from Guo et al. (2016). Species: *DS* *D. subspicatus*; *PS* *P. subcapitata*; *NP* *N. pelliculosa*; *AF* *A. flos-aquae*. Experimental conditions for algal test: 24 h illumination (76 µmol m^−2^ s^−1^), continuous shaking [100 cycles per minute (cpm)], fixed temperature (20 ± 2 °C) and 4-days exposure

While SPE was performed to concentrate the exposure solutions for the tests on *A. flos-aquae* before the algal testing, the volume of solution in the test vial at the end of the study was less than 15 mL so it was not possible to conduct SPE again. While no stability data of the antibiotics for studies with *A. flos-aquae* during the 4-days period are available, stability data of lincomycin and tylosin have been generated by us in a previous study (Guo et al. 2016), so these were applied to extrapolate to the intermediate concentration. Data on the stability of the study compounds during the tests on the two chlorophytes and the diatom are presented in Fig. 1. Stability varied depending on test concentration and species. For tylosin, concentrations at the end of the study ranged from 40.96 % (*N. pelliculosa* exposed to a concentration of 7.25 µmol L^−1^) to 129 % (*P. subcapitata* exposed to 0.4 µmol L^−1^) of the starting concentration. For lincomycin, the range of recovery was 33 % (*N. pelliculosa* exposed to a concentration of 225.73 µmol L^−1^) to 131.1 % (*D. subspicatus* exposed to 18.87 µmol L^−1^). For trimethoprim, the range was 12.75 % (*P. subcapitata* exposed to 30.69 µmol L^−1^) to 105.08 % (*N. pelliculosa* exposed to 146.32 µmol L^−1^). The recovery for each antibiotic during the 4-days test period is important as the significant losses of test compounds from the test system might result in an underestimation of their toxicity. The reductions in concentrations could be due to a range of processes, including abiotic (photolysis, hydrolysis) or biotic (i.e., metabolism by the algae) degradation or due to sorption or uptake to/into the algal cells. This subject has been thoroughly discussed in Guo et al. (2016) and will not be repeated here. The three antibiotics are known to be hydrolytically stable and resistant to biodegradation (Guo et al. 2016), so the disappearance of antibiotics is likely explained by a combination of photolysis, sorption and uptake to/into the algal cells, which have been previously reported in the literature (Di Paola et al. 2006; Sirtori et al. 2010; Mitchell et al. 2015; OECD 2011).

**Fig. 2:**
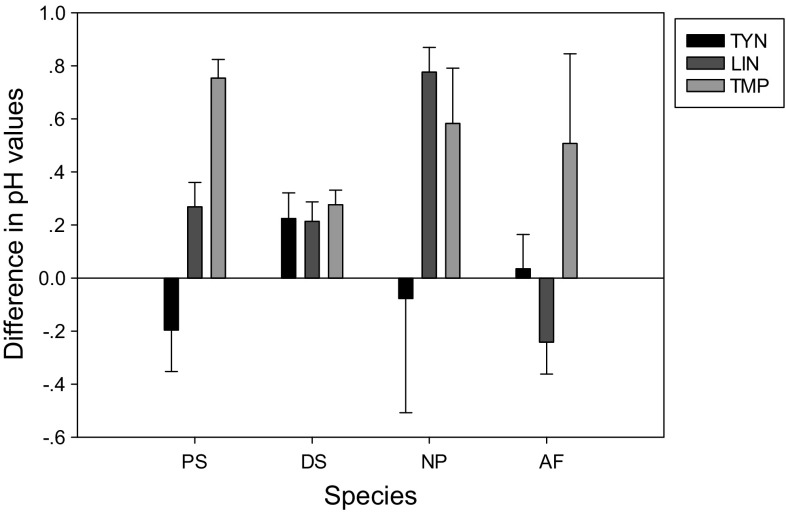
Changes in pH during 4 days of exposure to antibiotics. Data represent mean ± SD (*n* = 21). *PS* *P. subcapitata*; *DS* *D. subspicatus*; *NP N. pelliculosa*; *AF* *A. flos-aquae*; *TYN* tylosin; *LIN* lincomycin; *TMP* trimethoprim. Experimental conditions for algal test: 24 h illumination (76 µmol m^−2^s^−1^), continuous shaking [100 cycles per minute (cpm)], fixed temperature (20 ± 2 °C) and 4-days exposure

During an algal toxicity test, the pH value will usually increase (Luetzhoft et al. 1999; Halling-Sorensen 2000). An explanation is that CO_2_ mass transfer from the surrounding air could not fulfill the growth of algae due to the carbon demand of algal growth. CO_2_ was then derived from biocarbonate in the medium resulting in an increase in pH (Luetzhoft et al. 1999). In this study, there was no significant difference between the pH of the medium at the start and the end of the study for most tests (Fig. 2). The exceptions were tests with trimethoprim on *P. subcapitata*, *N. pelliculosa,* and *A. flos-aquae*, lincomycin on *N. pelliculosa,* and tylosin on *P. subcapitata* where a maximum increase of 0.8 units was observed; this value is within the variation considered acceptable by the OECD 201 guideline (<1.5 units). This result agreed with published work, e.g., in tests of trimethoprim on the chlorophyte *P. subcapitata* and cyanobacteria *A. flos-aquae*, the pH values increased from 7.6 to 8.3 and from 7.1 to 7.4, respectively (Kolar et al. 2014). An increase in pH can affect the toxicity of ionisable compounds, such as the study antibiotics. The pH values of the different algal media (6.8–8.2) would promote the ionisation of the tested antibiotics in solutions, which resulted in the neutral fractions ranging from 20.08 to 92.32 % (Table 1). Effects of antibiotic ionisation on algal toxicity and sensitivity have been thoroughly discussed in Guo et al. (2016) and therefore will not be repeated here. The readers should only have in mind that for acidic antibiotics, such as tylosin (Pka 7.73) and lincomycin (Pka 7.6), increasing pH values would lower their toxicity in algal tests by promoting ionisation of the antibiotics, which would reduce uptake into the cells (Halling-Sorensen 2000). For the weak base trimethoprim (Pka 7.12), an increasing pH would increase its toxicity by increasing the percentage of neutral compound.

**Table 1:** Summary of EC_50_ (µmol L^−1^) data based on two endpoints (growth and gross photosynthesis) for three antibiotics on four algal species over 4-days exposures

	Tylosin				Trimethoprim				Lincomycin			
	Growth	Photosynthesis	pH range	Neutral fraction (%)	Growth	Photosynthesis	pH range	Neutral fraction (%)	Growth	Photosynthesis	pH range	Neutral fraction (%)
DS	38.27 (30.23–47.08)	17.6 (10.13–13.39)	6.65–7.76	89.49	>272.7	>272.7	5.99–6.31	32.37	>188.71	79.41 (60.27–103.3)	7.38–7.8	86.32
PS	4.8 (4.26–5.47)	2.1 (n.a.)	6.69–6.86	89.49	>307	>307	6.77–7.03	32.37	24.14 (21.84–27.6)	12 (n.a.–20.68)	5.92–6.06	86.32
AF	0.06 (n.a.–0.068)	0.33 (0.21–0.52)	6.99–8.04	45.98	>341.69	>341.69	7.21–7.85	82.72	1.2 (1.04–1.51)	4.75 (0.49–n.a.)	7.28–7.78	38.69
NP	4.4 (3.66–5.05)	7.35 (0.44–17.49)	7.75–8.36	25.31	70.48 (57.79–96.03)	136.36 (95.34–n.a.)	8.54–9.1	92.32	>153.91	>153.91	8.81–9.07	20.08

Numbers in brackets indicate 95% confidence limits

*n.a*. not available

Species: *DS* *D. subspicatus*; *PS* *Psubcapitata*; *AF* *A. flos-aquae*; *NP* *N pelliculosa*

EC_50_ values for the reference toxicant, potassium dichromate on two chlorophytes, *D. subspicatus* and *P. subcapitata,* were 4.59 and 5.23 µmol L^−1^, respectively. These results are consistent with previously reported data where the EC_50_ for the substance was found to range from 1.33 to 4.86 µmol L^−1^ for *D. subspicatus* and 1.29–8.89 µmol L^−1^ for *P. subcapitata* (Pattard 2009). The EC_50_ found for diatom *N. pelliculosa* and *A. flos-aquae* were >33.99 and 15.94 µmol L^−1^, respectively. However, no information on the toxicity of potassium dichromate to these two species is available in the literature for comparison purposes.

### Endpoint Sensitivity Comparison

All the exposure concentrations used for plotting concentration–response curves have been revised using modified chemical recoveries (Supplemental data). While this study characterised the inhibition effects of antibiotics on the pigment synthesis, the results of pigment content (total chlorophyll content and carotenoids) after 4-days exposure could not be fitted to concentration–response curves. Therefore, it was only possible to derive concentration–response curves based on effects on growth and oxygen evolution rate to derive EC_50_ values. These data are described in the next section along with a discussion of the sensitivity of the different endpoints.

#### Toxicity Test Analysis Based on Growth

Studies into the effects of the three study antibiotics on the growth of a selection of algal species have been reported previously. In our study the 96 h EC_50_ for tylosin for growth inhibition of *P. subcapitata* was 4.8 µmol L^−1^ (Table 1), which agrees with the previous studies where 72 h EC_50_ values have been reported to range from 0.38 to 1.51 µmol L^−1^ (Halling-Sorensen 2000; Eguchi et al. 2004). For *A. flos-aquae,* we obtained a 96 h EC_50_ of 0.06 µmol L^−1^, which is within an order of magnitude of a published EC_50_ of 0.037 µmol L^−1^, which was reported for another cyanobacterial species, *Microcystis aeruginosa,* after 72-h exposure to tylosin (Halling-Sorensen 2000). The 96-h EC_50_ for lincomycin for *A. flos-aquae* growth inhibition was 1.2 µmol L^−1^; this is not dissimilar to the 96 h EC_50_ value reported for the cyanobacteria *Synechococcus leopoliensis* of 0.49 µmol L^−1^ (Andreozzi et al. 2006). The 96-h EC_50_ for lincomycin to the chlorophyte *P. subcapitata* was 24.14 µmol L^−1^ (Table 1), which is higher than previously reported values for the same species 3.71 µmol L^−1^ (96-h EC_50_) (Andreozzi et al. 2006).

There are numerous explanations for variations between our data and previous studies, including differences in test conditions (e.g., in initial inoculation cell number) or differences in the sensitivities of individual species within an algal class. As suggested by OECD 201 guideline (OECD 2011), low cell numbers ranging from 5 × 10^3^ to 5 × 10^4^ cells mL^−1^ were usually used for pure toxicity tests (van der Grinten et al. 2010; Andreozzi et al. 2006). In this study, the inoculated cell number was set at 5 × 10^5^ cells mL^−1^ to allow the oxygen evolution rate to be measured after the 4-days exposure. A higher initial cell number could ensure that the oxygen evolution rates of algal cultures are above the limit of detection of the DW2 Oxygen Electrode Chamber. However, a higher initial cell density could lead to less toxicant content bonding to the cells (both intercellular and extracellular) and further lead to less toxicant uptake and lowering of toxicity (Franklin et al. 2002). This trend has been reported in tests with copper on the chlorophyte *P. subcapitata*, where significantly more extra- and intracellular copper was accumulated at algal initial cell density at 10^3^ cells mL^−1^ compared to 10^4^ and 10^5^ cells mL^−1^ for the medium with the same copper concentration. The toxicity at 72 h EC_50_ level in terms of growth rate significantly decreased from 97.56 to 118.02 and 267.51 µmol L^−1^ as cell density increased (Franklin et al. 2002). Despite previous studies showing lincomycin to affect the diatom *Cyclotella meneghiniana* with a reported 96-h EC_50_ of 4 µmol L^−1^ (Andreozzi et al. 2006), in the current study, no effect was found for the diatom *N. pelliculosa* at the top test concentration of 153.91 µmol L^−1^. Potential effects of trimethoprim were recorded for the chlorophyte *P. subcapitata* (72 h EC_50_ 276.59–444.34 µmol L^−1^) (Eguchi et al. 2004; Kolar et al. 2014) and cyanobacteria *A. flos-aquae* (72 h EC_50_ 871.45 µmol L^−1^; Kolar et al. 2014), which agreed with the results of this study (>307 µmol L^−1^ for *P. subcapitata* and >341.69 for *A. flos-aquae*; Table 1). The 96-h EC_50_ for trimethoprim for the diatom *N. pelliculosa* was 70.48 µmol L^−1^; this compound does not appear to have been tested previously on diatoms.

#### Toxicity Test Analysis Based on Photosynthesis and Endpoint Sensitivity Comparison

For the two chlorophytes, photosynthesis was found to be a more sensitive endpoint than growth. After 4-days exposure to tylosin, the EC_50_ values for the two chlorophytes, *D. subspicatus* and *P. Subcapitata,* based on photosynthesis as an endpoint were 17.6 and 2.1 µmol L^−1^, respectively. Similar results were observed for two chlorophytes exposed to lincomycin (Table 1). However, for cyanobacteria *A. flos-aquae* and diatom *N. pelliculosa*, the situation was reversed and growth appeared to be a more sensitive endpoint than photosynthesis (Table 1). For example, after 4-days exposure of *A. flos-aquae* to lincomycin, the EC_50_ derived based on growth was 1.2 µmol L^−1^ (Table 1), which was nearly one third of that derived based on photosynthesis. While no explanation for the sensitivity behaviour of both endpoints was available, the results of this study indicated that when testing antibiotics on chlorophytes for the environmental risk assessment purpose, oxygen evolution rate measurements might be an additional endpoint that could be included, which, in some cases, may be more sensitive as well a being ecologically relevant as photosynthesis is such an important process for ecosystem functioning.

### Analysis of the Toxic Effects on the Algal Physiology

#### Toxic Effects on the Oxygen Evolution Rate

All three antibiotics significantly inhibited the oxygen evolution rate of gross photosynthesis (Table 2). The inhibition effects were increased with the increasing concentrations of antibiotics. For example, the gross photosynthesis rate of *P. subcapitata* treated with tylosin at the concentrations of 3.61 and 9.12 µmol L^−1^ were 0.052 unit (µmol O_2_ h^−1^ cell^−1^ 10^6^) and 0.023 unit, respectively, which only account for 26 and 11.5 % of that in control. This result agreed with the literature. Liu et al. (2011) reported that after 4-days exposure to macrolide erythromycin at the concentrations of 0.16 and 0.33 µmol L^−1^, the photosynthetic oxygen evolution rate of a same species decreased from 372.89 unit (µmol O_2_ min^−1^ g^−1^ fresh weight) in control to 195.46 units and 112.3 units. Antibiotics do not affect the algal gross photosynthesis and pigment synthesis at the same concentration level, e.g. after 4-days exposure to lincomycin at the concentration of 18.87 µmol L^−1^, whereas the gross photosynthesis rate of *D. subspicatus* decreased from 0.46 unit in control to 0.34 unit; no evident reduction in total chlorophyll and carotenoid contents were observed (Table 2). A similar result was reported in the study by Hudock et al. (1964) testing a different toxicant chloramphenicol. It was found that the chlorophyte *Chlamydomonas reinhardi* treated with 61.89 µmol L^−1^ chloramphenicol would inhibit the oxygen evolution rate but had no effect on chlorophyll content. They inferred that the photosynthesis rate was not correlated with a factor directly related to chlorophyll synthesis (Hudock et al. 1964).

**Table 2:** Values of net photosynthesis, respiration, gross photosynthesis rate, total chlorophyll content, and carotenoid content per cell of *D. subspicatus*, *P. subcapitata*, *N. pelliculosa*, and *A. flos-aquae* over 4-days antibiotic exposures for three antibiotics: tylosin, trimethoprim, and lincomycin

Algae	Antibiotic	4 days TWAC (µmol L^−1^)	Net photosynthesis/cells (µmol O_2_ h^−1^ cell^−1^ 10^6^)	Respiration/cells (µmol O_2_ h^−1^ cell^−1^ 10^6^)	Gross photosynthesis /cells (µmol O_2_ h^−1^ cell^−1^ 10^6^)	Total chlorophyll/cell (10^9^ mg L^−1^ cell^−1^)	Total carotenoid/cells (10^9^ mg L^−1^ cell^−1^)
*D. subspicatus*	Tylosin	Control	0.233 ± 0.108	−0.27 ± 0.077	0.507 ± 0.045	2.4 ± 0.31	0.59 ± 0.073
		6.49	0.282 ± 0.067	−0.16 ± 0.083	0.44 ± 0.027	2.32 ± 0.95	0.56 ± 0.204
		12.99	0.339 ± 0.028	−0.11 ± 0.011*	0.45 ± 0.018	2.38 ± 0.29	0.60 ± 0.066
		25.97	0.092 ± 0.022	−0.0058 ± 0.018*	0.097 ± 0.016*	2.88 ± 1.01	0.72 ± 0.202
		42.94	0.074 ± 0.037*	−0.051 ± 0.033*	0.125 ± 0.039*	1.82 ± 0.17	0.49 ± 0.042
		57.26	0.093 ± 0.091*	−0.093 ± 0.077*	0.185 ± 0.12*	1.67 ± 0.45	0.45 ± 0.107
		71.56	0.076 ± 0.0085*	−0.045 ± 0.039*	0.12 ± 0.048*	2.37 ± 0.27	0.61 ± 0.07
	lincomycin	Control	0.38 ± 0.031	−0.076 ± 0.024	0.46 ± 0.055	3 ± 0.44	0.71 ± 0.1
		18.87	0.25 ± 0.031*	−0.092 ± 0.0068	0.34 ± 0.035*	2.95 ± 0.25	0.72 ± 0.063
		37.74	0.19 ± 0.047*	−0.112 ± 0.016	0.304 ± 0.034*	3.56 ± 0.49	0.88 ± 0.12
		75.49	0.11 ± 0.054*	−0.11 ± 0.0072	0.22 ± 0.053*	2.45 ± 0.52	0.63 ± 0.12
		113.23	0.07 ± 0.05*	−0.13 ± 0.014*	0.2 ± 0.041*	2.72 ± 0.19	0.69 ± 0.037
		150.97	0.027 ± 0.015*	−0.14 ± 0.035*	0.17 ± 0.023*	3.05 ± 0.24	0.78 ± 0.07
		188.71	0.02 ± 0.018*	−0.11 ± 0.015	0.13 ± 0.0046*	2.5 ± 0.22	0.64 ± 0.067
	Trimethoprim	Control	0.23 ± 0.11	−0.19 ± 0.01	0.43 ± 0.1	3.02 ± 0.17	0.72 ± 0.039
		27.25	0.2 ± 0.16	−0.14 ± 0.0063	0.34 ± 0.16	2.3 ± 0.32	0.57 ± 0.069
		54.53	0.25 ± 0.13	−0.18 ± 0.034	0.43 ± 0.16	2.41 ± 0.24	0.59 ± 0.045
		109.09	0.24 ± 0.13	−0.2 ± 0.019	0.44 ± 0.14	2.65 ± 0.46	0.65 ± 0.089
		163.61	0.31 ± 0.11	−0.18 ± 0.033	0.49 ± 0.13	2.64 ± 0.63	0.66 ± 0.15
		218.14	0.3 ± 0.088	−0.15 ± 0.053	0.45 ± 0.13	2.88 ± 0.54	0.703 ± 0.12
		272.7	0.36 ± 0.033	−0.15 ± 0.033	0.51 ± 0.066	2.25 ± 0.18	0.56 ± 0.051
*P. subcapitata*	Tylosin	Control	0.086 ± 0.055	−0.11 ± 0.023	0.2 ± 0.046	0.745 ± 0.18	0.2 ± 0.042
		0.4	0.098 ± 0.045	−0.095 ± 0.013	0.19 ± 0.052	0.746 ± 0.15	0.21 ± 0.03
		1.2	0.1 ± 0.038	−0.098 ± 0.012	0.2 ± 0.045	0.749 ± 0.081	0.205 ± 0.024
		3.61	−0.08 ± 0.006*	−0.13 ± 0.027	0.052 ± 0.03*	0.82 ± 0.063	0.23 ± 0.026
		9.12	−0.2 ± 0.045*	−0.22 ± 0.04	0.023 ± 0.006*	1.24 ± 0.16	0.307 ± 0.041
		18.23	−0.3 ± 0.095*	−0.32 ± 0.092*	0.012 ± 0.006*	2.12 ± 0.13*	0.53 ± 0.036*
		27.35	−0.32 ± 0.083*	−0.33 ± 0.083*	0.008 ± 0.006*	0.81 ± 0.17*	0.19 ± 0.068*
	Lincomycin	Control	0.073 ± 0.036	−0.063 ± 0.0078	0.136 ± 0.039	0.44 ± 0.049	0.14 ± 0.014
		17	−0.029 ± 0.022*	−0.082 ± 0.023	0.053 ± 0.007	0.51 ± 0.204	0.18 ± 0.069
		34	−0.055 ± 0.01*	−0.096 ± 0.037	0.041 ± 0.044	0.58 ± 0.22	0.19 ± 0.056
		68	−0.078 ± 0.014*	−0.089 ± 0.014	0.0107 ± 0.002	0.78 ± 0.3	0.23 ± 0.069
		125	−0.104 ± 0.032*	−0.11 ± 0.032	0.0073 ± 0.002*	0.7 ± 0.28	0.214 ± 0.071
		166.61	−0.124 ± 0.039*	−0.13 ± 0.035*	0.0069 ± 0.005*	0.36 ± 0.21	0.12 ± 0.05
		208.28	−0.131 ± 0.014*	−0.14 ± 0.016*	0.0052 ± 0.002*	0.85 ± 0.86	0.23 ± 0.18
	Trimethoprim	Control	0.044 ± 0.022	−0.073 ± 0.0205	0.117 ± 0.034	0.88 ± 0.13	0.26 ± 0.03
		13.2	0.058 ± 0.038	−0.059 ± 0.014	0.017 ± 0.04	0.707 ± 0.054	0.208 ± 0.013
		26.42	0.059 ± 0.036	−0.063 ± 0.023	0.12 ± 0.046	0.908 ± 0.15	0.26 ± 0.038
		52.83	0.058 ± 0.014	−0.073 ± 0.015	0.13 ± 0.024	0.97 ± 0.13	0.28 ± 0.025
		103.29	0.05 ± 0.015	−0.07 ± 0.018	0.12 ± 0.023	0.818 ± 0.039	0.24 ± 0.002
		137.73	0.043 ± 0.01	−0.068 ± 0.022	0.11 ± 0.025	0.928 ± 0.086	0.27 ± 0.013
		172.15	0.033 ± 0.006	−0.072 ± 0.019	0.1 ± 0.022	0.801 ± 0.093	0.23 ± 0.023
*N. pelliculosa*	Tylosin	Control	0.071 ± 0.016	−0.081 ± 0.013	0.15 ± 0.014	0.74 ± 0.053	0.502 ± 0.041
		4.88	−0.01 ± 0.012*	−0.1 ± 0.061	0.086 ± 0.051*	0.86 ± 0.1	0.64 ± 0.067
		9.77	−0.04 ± 0.009*	−0.11 ± 0.013	0.07 ± 0.012*	1.05 ± 0.12	0.8 ± 0.089
		19.53	−0.06 ± 0.019*	−0.11 ± 0.011	0.051 ± 0.017*	1.1 ± 0.2	0.85 ± 0.19
		41.72	−0.06 ± 0.007*	−0.096 ± 0.02	0.032 ± 0.021*	1.05 ± 0.34	0.8 ± 0.28
		59.6	−0.06 ± 0.02*	−0.099 ± 0.04	0.036 ± 0.03*	1.24 ± 0.4	0.95 ± 0.32
		77.4	−0.06 ± 0.014*	−0.12 ± 0.022	0.054 ± 0.023*	1.34 ± 0.17	1.06 ± 0.13*
	Lincomycin	Control	0.02 ± 0.023	−0.12 ± 0.025	0.14 ± 0.037	0.76 ± 0.18	0.56 ± 0.14
		21.44	0.031 ± 0.022	−0.089 ± 0.004	0.12 ± 0.021	0.56 ± 0.024	0.42 ± 0.023
		42.88	0.035 ± 0.023	−0.09 ± 0.031	0.13 ± 0.051	0.73 ± 0.24	0.5 ± 0.13
		64.33	0.026 ± 0.014	−0.1 ± 0.027	0.13 ± 0.041	0.77 ± 0.19	0.58 ± 0.14
		82.03	0.048 ± 0.007	−0.1 ± 0.031	0.15 ± 0.038	0.64 ± 0.1	0.38 ± 0.1
		102.61	0.05 ± 0.009	−0.095 ± 0.022	0.15 ± 0.031	1.03 ± 0.34	0.77 ± 0.24
		153.91	0.053 ± 0.027	−0.093 ± 0.007	0.15 ± 0.033	0.78 ± 0.2	0.58 ± 0.17
	Trimethoprim	Control	0.026 ± 0.016	−0.17 ± 0.047	0.19 ± 0.032	0.57 ± 0.096	0.43 ± 0.077
		10.85	0.035 ± 0.009	−0.16 ± 0.02	0.19 ± 0.013	0.66 ± 0.038	0.49 ± 0.031
		16.26	0.026 ± 0.004	−0.16 ± 0.03	0.19 ± 0.034	0.6 ± 0.053	0.45 ± 0.031
		32.52	0.059 ± 0.012	−0.16 ± 0.04	0.22 ± 0.042	0.7 ± 0.04	0.51 ± 0.039
		48.77	−0.01 ± 0.016	−0.18 ± 0.085	0.17 ± 0.075	0.63 ± 0.048	0.49 ± 0.033
		97.55	−0.15 ± 0.061*	−0.29 ± 0.101	0.14 ± 0.041	1.68 ± 0.6*	1.4 ± 0.48*
		146.32	−0.19 ± 0.068*	−0.27 ± 0.051	0.086 ± 0.023*	1.14 ± 0.2*	0.97 ± 0.17*
*A. flos-aquae*	Tylosin	Control	0.058 ± 0.041	−0.1 ± 0.0093	0.16 ± 0.05	0.26 ± 0.032	0.194 ± 0.034
		0.032	0.07 ± 0.039	−0.097 ± 0.17	0.17 ± 0.051	0.24 ± 0.062	0.166 ± 0.052
		0.064	0.03 ± 0.014	−0.074 ± 0.033	0.1 ± 0.019	0.27 ± 0.033	0.215 ± 0.024
		0.19	−0.1 ± 0.026*	−0.19 ± 0.031	0.092 ± 0.034	0.24 ± 0.002	0.191 ± 0.007
		0.5	−0.18 ± 0.084*	−0.21 ± 0.065	0.034 ± 0.054*	0.4 ± 0.064	0.332 ± 0.045
		1.06	−0.18 ± 0.091*	−0.18 ± 0.051	−0.0032 ± 0.042*	0.47 ± 0.178*	0.366 ± 0.137*
		2.11	−0.2 ± 0.073*	−0.2 ± 0.095	−0.0071 ± 0.022*	0.49 ± 0.048*	0.384 ± 0.038*
	Lincomycin	Control	0.028 ± 0.025	−0.13 ± 0.023	0.16 ± 0.026	0.35 ± 0.137	0.25 ± 0.095
		0.12	−0.01 ± 0.034	−0.14 ± 0.013	0.13 ± 0.034	0.56 ± 0.267	0.363 ± 0.14
		0.23	−0.04 ± 0.007	−0.144 ± 0.016	0.104 ± 0.021	0.31 ± 0.112	0.227 ± 0.074
		1.38	−0.11 ± 0.042*	−0.21 ± 0.078	0.101 ± 0.054	0.47 ± 0.146	0.35 ± 0.091
		2.93	−0.17 ± 0.054*	−0.25 ± 0.087	0.079 ± 0.034*	0.83 ± 0.176*	0.57 ± 0.113*
		5.87	−0.17 ± 0.065*	−0.25 ± 0.08	0.08 ± 0.02*	0.6 ± 0.05	0.43 ± 0.035
	Trimethoprim	Control	0.091 ± 0.019	−0.066 ± 0.034	0.16 ± 0.035	0.27 ± 0.046	0.18 ± 0.032
		23.21	0.094 ± 0.055	−0.064 ± 0.027	0.16 ± 0.03	0.22 ± 0.016	0.14 ± 0.008
		46.42	0.056 ± 0.056	−0.078 ± 0.0098	0.13 ± 0.065	0.22 ± 0.015	0.13 ± 0.008*
		92.83	0.085 ± 0.034	−0.067 ± 0.023	0.15 ± 0.052	0.22 ± 0.041	0.12 ± 0.029*
		205.02	0.084 ± 0.057	−0.067 ± 0.021	0.15 ± 0.073	0.27 ± 0.029	0.17 ± 0.02
		273.35	0.101 ± 0.025	−0.067 ± 0.018	0.168 ± 0.041	0.23 ± 0.025	0.14 ± 0.017
		341.69	0.069 ± 0.019	−0.064 ± 0.017	0.13 ± 0.035	0.24 ± 0.041	0.15 ± 0.013

Data are presented as mean values ± standard deviation (*n* = 3); *Asterisks* indicate significant difference

#### Toxic Effects on Pigment Synthesis

Exposure to three antibiotics could result in reduction in total chlorophyll and carotenoid contents of test algal species e.g. the chlorophyll of *D. subspicatus* decreased from 2.4 units (10^9^ mg L^−1^ cell^−1^) in control to 1.67 units after 4-days exposure to tylosin at the concentration of 57.26 µmol L^−1^, and simultaneously the total carotenoid reduced from 0.59 unit (10^9^ mg L^−1^ cell^−1^) to 0.45 unit (Table 2). These observed inhibitory effects of the macrolide on algal pigment synthesis agreed with a study by Liu et al. (2011). It was reported that the macrolide erythromycin, at a concentration of 0.41 µmol L^−1^, results in a reduction in the chlorophyll content of *P. subcapitata* to 0.4 mg g^−1^ fresh weight in contrast with 0.95 mg g^−1^ in the control. However, in some cases, pigment contents were stimulated for *P. subcapitata*, *N. pelliculosa* and *A. flos-aquae* at some concentration levels (Table 2). For example, after 4d exposure to tylosin at 18.23 µmol L^−1^, total chlorophyll content and carotenoid per cell of *P. subcapitata* increased by 185 and 165 % compared to that in control. Similar stimulation effects have been reported by studies testing other toxicants (polyamidoamine (PAMAM) 1,4-diaminobutane core, G2), where total chlorophyll content increased by 121 % compared with the control at a concentration of 0.76 µmol L^−1^ (Petit et al. 2010). In the literature, a few of studies only present the measured pigment contents in the unit of mg L^−1^, without correction for cell density or weight. For example, the carotenoid content of the prokaryote *Sarcina lutea* was reduced from 63 mg L^−1^ in the control to 38 mg L^−1^ over 1-day exposure to 14.24 µmol L^−1^ chloramphenicol (Portoles et al. 1970). In this case, the reduction in pigment might be attributed to less algae existing in the solution due to reduced growth.

#### Toxic Effects on the Irradiance–Photosynthesis Relationship

**Fig. 3:**
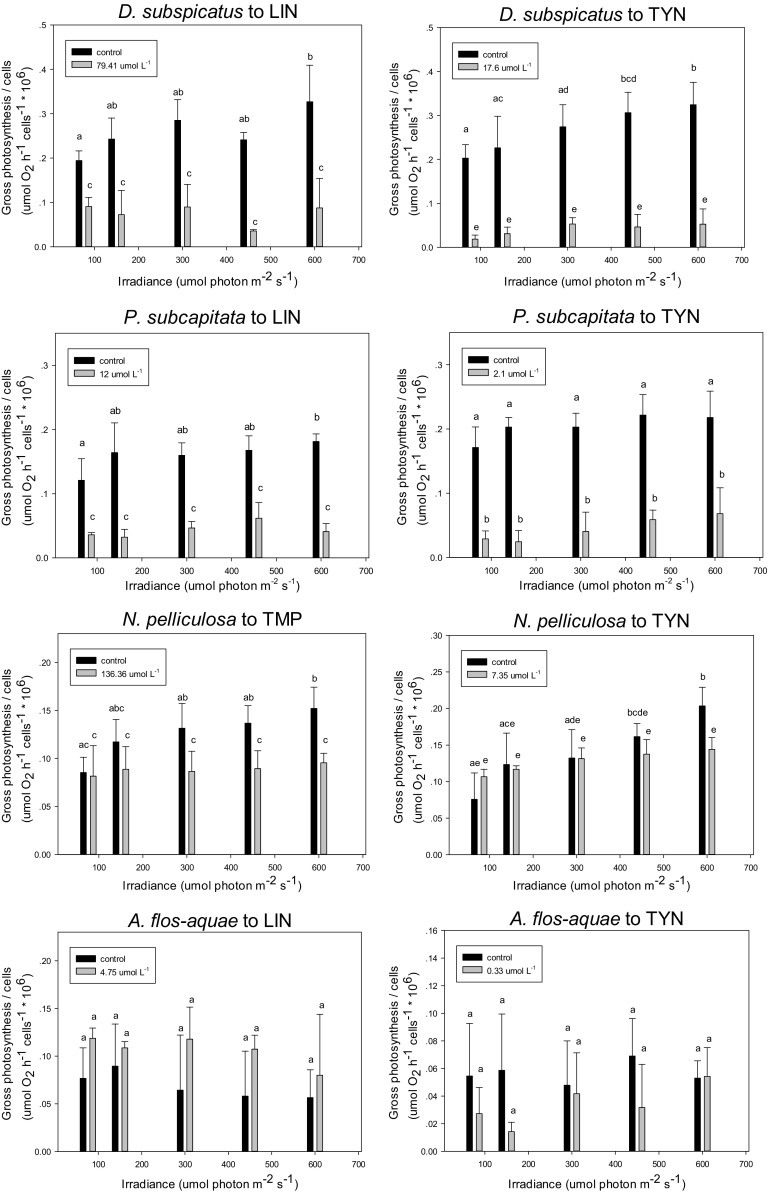
Responses of the gross photosynthetic rate on irradiance for algal species with evident photosynthesis inhibition effect from antibiotics. Data represent mean ± SD (*n* = 3). Bars sharing the same letter code are not significantly different; *LIN* lincomycin; *TYN* tylosin; *TMP* trimethoprim. Experimental conditions for algal test: 24-h illumination (76 µmol m^−2^ s^−1^), continuous shaking [100 cycles per minute (cpm)], fixed temperature (20 ± 2 °C) and 4-days exposure

The gross oxygen evolution rate in the control cultures of *D. subspicatus*, *P. subcapitata,* and *N. pelliculosa* increased with increasing irradiance level and the trend followed a typical irradiance–photosynthesis (I–P) curve (Fig. 3), where significant differences between controls and treated samples were observed for these species. While the oxygen evolution rate in the treated samples exhibited a similar increasing trend, each evolution rate was still lower than that of the control (except for *A. flos-aquae*). The gap of gross oxygen evolution rate between control and treated samples was enlarged with higher irradiance. For example, with an increase in the light intensity from 76 to 600 µmol m^−2^ s^−1^, whereas the gross photosynthesis rate (*P*_g_) of *D. subspicatus* in treatment raised from 0.019 unit (µmol O_2_ h^−1^ cell^−1^ 10^6^) to 0.053 unit, *P*_g_ values in controls increased from 0.2 unit to 0.32 unit. However, in the cyanobacteria *A. flos-aquae*, no significant differences between controls and treated samples were observed, though EC_50_s of lincomycin and tylosin based on photosynthesis were applied. The reason might be due to that the EC_50_ derived was not significantly different. For example, after 4-days exposure to tylosin, EC_50_ derived from concentration–response curve (gross oxygen evolution rate) was 0.33 µmol L^−1^, which was lower than the lowest-observed-effect- concentration (LOEC, 0.5 µmol L^−1^; Table 2). No increasing trend of oxygen evolution rate was shown with increasing irradiance as light has already achieved saturation or higher (Fig. 3). These findings agreed with other published work; Bahrs et al. (2013) found that significant differences in P–I relationship could be observed for the chlorophyte *Desmodesmus armatus* and the cyanobacteria *Synechocystis* sp. between the control and samples treated with polyphenol *p*-benzoquinone at the EC_90_ level based on growth. In particular, the maximum gross oxygen production of *Synechocystis* sp. in treated sample was five times lower than that in the control. However, no significant effects of *p*-benzoquinone were found on the P–I relation of cyanobacteria *Microcystis aeruginosa*.

## Conclusions

This study indicated that after 4-days exposure to antibiotics tylosin, lincomycin, and trimethoprim, the photosynthesis related endpoint (oxygen evolution rate) exhibited higher sensitivity than the growth endpoint in the test with chlorophytes. The situation was reversed when testing antibiotics on cyanobacteria and diatoms. It is recommended that more species from each class should be involved in testing antibiotics to confirm this conclusion. Once the verdict has been confirmed, in addition to the endpoint of growth, oxygen evolution rate might be an endpoint that could be used in the future regulatory ecotoxicity studies. This study revealed that antibiotics inhibit the pigment synthesis in some algal species (e.g., *D. subspicatus*), although the stimulation effects were also observed. While the light utilization efficiency of eukaryote chlorophytes and diatom are reduced after exposure to the antibiotics, no significant inhibition effect on prokaryote cyanobacteria was observed. As algal species are of importance in the aquatic environment due to their ecological functions, such as primary production and nutrient transformation, adverse effects of antibiotic on algae will impact the ecosystem.

## Electronic supplementary material

Below is the link to the electronic supplementary material.

Supplementary material 1 (DOCX 46 kb)
