# Supplementary material for: Effects of Antibiotics on the Growth and Physiology of Chlorophytes, Cyanobacteria, and a Diatom
Source: Arch Environ Contam Toxicol. 2016 Aug 9;71(4):589–602. doi: 10.1007/s00244-016-0305-5 (PMC5566184; doi:10.1007/s00244-016-0305-5)
Supplement: Supplementary file 1 — Supplementary material 1 (DOCX 46 kb) [file 244_2016_305_MOESM1_ESM.docx]

**Supplemental data**

Table 1: Extrapolation of chemical recovery (%) for each time points (d). DS, *D. subspicatus*; PS, *P. subcapitata*; NP, *N. pelliculosa*; AF, *A. flos-aquae*.

| Tylosin | Chemical recovery (%) | | | |
| --- | --- | --- | --- | --- |
|  | 1d | 2d | 3d | 4d |
| Low |  |  |  |  |
| DS | 94.19 | 88.82 | 83.86 | 79.27 |
| NP | 90.18 | 81.63 | 74.15 | 67.61 |
| AF | 89.54 | 80.51 | 72.7 | 65.91 |
| High |  |  |  |  |
| PS | 95.67 | 91.59 | 87.74 | 84.11 |
| DS | 96.62 | 93.4 | 90.32 | 87.37 |
| NP | 95.2 | 90.7 | 86.48 | 82.53 |
| AF | 96.46 | 93.09 | 89.87 | 86.81 |
| Lincomycin |  |  |  |  |
| Low |  |  |  |  |
| PS | 94.92 | 90.18 | 85.76 | 81.63 |
| NP | 94.15 | 88.75 | 83.77 | 79.16 |
| High |  |  |  |  |
| NP | 90.39 | 82 | 74.64 | 68.18 |
| Trimethoprim |  |  |  |  |
| Low |  |  |  |  |
| PS | 78.57 | 63.03 | 51.59 | 43.03 |
| AF | 90.3 | 81.83 | 74.42 | 67.92 |
| High |  |  |  |  |
| PS | 85.43 | 73.62 | 63.98 | 56.07 |

Table 2: Regression models used to derive concentration-response curves for each antibiotic. DS, *D. subspicatus*; PS, *P. subcapitata*; NP, *N. pelliculosa*; AF, *A. flos-aquae*.

| Chemicals | species | Equation based on the endpoint of growth | Parameters | Equation based on the endpoint of photosynthesis | Parameter |
| --- | --- | --- | --- | --- | --- |
| Lincomycin | PS | **Logistic** f = if(x<=0, if(b<0,0,a), if(b>0, a/(1+abs(x/x0)^b), a*abs((x/x0))^(abs(b))/(1+(abs(x/x0))^(abs(b))))) R^2^=0.99 | a=85.8243  b=-1.5259  c=19.5198 | **Hill** f = a*x^b/(c^b+x^b) R^2^=0.84 | A=103.429  B=0.9965  C=12.7343 |
|  | DS | **Hill** f = a*x^b/(c^b+x^b) R^2^=0.89 | a=56.1954  b=1.1138  c=37.3791 | **Chapman** f = a*(1-exp(-b*x))^c R^2^=0.92 | A=101.6327  B=0.0035  C=0.501 |
|  | AF | **Weibull** f = if(x<=x0-b*ln(2)^(1/c), 0, a*(1-exp(-(abs(x-x0+b*ln(2)^(1/c))/b)^c))) R^2^=0.95 | A=65.8625  B=2.1045  C=3.5157  X0=0.7774 | **Hill** f = a*x^b/(c^b+x^b) R^2^=0.4 | A=56.5253  B=0.6066  C=0.1657 |
|  | NP | n.a | n.a | n.a | n.a |
| Tylosin | PS | **Weibull** f = if(x<=x0-b*ln(2)^(1/c), 0, a*(1-exp(-(abs(x-x0+b*ln(2)^(1/c))/b)^c))) R^2^=0.99 | a=82.5802  b=4.5983  c=1.1741  x0=3.855 | **Weibull** f = if(x<=x0-b*ln(2)^(1/c), 0, a*(1-exp(-(abs(x-x0+b*ln(2)^(1/c))/b)^c))) R^2^=0.91 | a=94.2789  b=1.366  c=0.6643  d=1.9873 |
|  | DS | **Weibull** f = if(x<=x0-b*ln(2)^(1/c), 0, a*(1-exp(-(abs(x-x0+b*ln(2)^(1/c))/b)^c))) R^2^=0.92 | a=96.6334  b=55.9902  c=0.833  x0=35.9337 | **Sigmoid** f = a/(1+exp(-(x-x0)/b)) R^2^=0.74 | A=67.5547  B=2.6127  X0=14.8269 |
|  | AF | **Chapman** f = a*(1-exp(-b*x))^c R^2^=0.95 | a=75.3458  b=72.5115  c=29.2359 | **Gompertz** f = a*exp(-exp(-(x-x0)/b)) R^2^=0.76 | A=103.8338  B=0.2401  C=0.2535  D=0.2352 |
|  | NP | **Logistic** f = if(x<=0, if(b<0,0,a), if(b>0, a/(1+abs(x/x0)^b), a*abs((x/x0))^(abs(b))/(1+(abs(x/x0))^(abs(b))))) R^2^=0.99 | a=73.3678  b=-1.0844  c=2.1734 | **Chapman** f = a*(1-exp(-b*x))^c R^2^=0.73 | A=73.3619  B=0.1006  C=0.5764 |
| Trimethoprim | PS | n.a | n.a | n.a | n.a |
|  | DS | n.a | n.a | n.a | n.a |
|  | AF | n.a | n.a | n.a | n.a |
|  | NP | **Chapman** f = a*(1-exp(-b*x))^c R^2^= 0.94 | a=70.4873  b=0.0276  c=2.4677 | **Chapman** f = a*(1-exp(-b*x))^c R^2^= 0.54 | A=98.6262  B=0.0152  C=5.0824 |
